# Efficacy of Eye Movement Desensitization and Reprocessing in Children and Adolescent with Post-traumatic Stress Disorder: A Meta-Analysis of Randomized Controlled Trials

**DOI:** 10.3389/fpsyg.2017.01750

**Published:** 2017-10-10

**Authors:** Ana Moreno-Alcázar, Devi Treen, Alicia Valiente-Gómez, Albert Sio-Eroles, Víctor Pérez, Benedikt L. Amann, Joaquim Radua

**Affiliations:** ^1^Institut de Neuropsiquiatria i Addiccions, Centre Fòrum Research Unit, Parc de Salut Mar, Barcelona, Spain; ^2^Hospital del Mar Medical Research Institute (IMIM), Barcelona, Spain; ^3^Centre Emili Mirà, Institut de Neuropsiquiatria i Addiccions, Parc de Salut Mar, Barcelona, Spain; ^4^Hospital Benito Menni Complex Assistencial en Salut Mental, Sant Boi del Llobregat, Spain; ^5^Department of Psychiatry, Autonomous University of Barcelona, Bellaterra, Spain; ^6^Mental Health Research Networking Center, Centro de Investigacion Biomedica en Red de Salud Mental, Madrid, Spain; ^7^FIDMAG Germanes Hospitalàries Research Foundation, Sant Boi del Llobregat, Spain; ^8^Centre for Psychiatric Research and Education, Department of Clinical Neuroscience, Karolinska Institutet, Stockholm, Sweden; ^9^Division of Psychosis Studies, Institute of Psychiatry, Psychology and Neuroscience, King’s College London, London, United Kingdom

**Keywords:** post-traumatic stress disorder, psychological trauma, EMDR, children, adolescents, meta-analysis

## Abstract

**Background:** Post-traumatic stress disorder (PTSD) can occur in both adults and children/adolescents. Untreated PTSD can lead to negative long-term mental health conditions such as depression, anxiety, low self-concept, disruptive behaviors, and/or substance use disorders. To prevent these adverse effects, treatment of PTSD is essential, especially in young population due to their greater vulnerability. The principal aim of this meta-analysis was to examine the efficacy of eye movement desensitization and reprocessing (EMDR) therapy for PTSD symptoms in children and adolescents. Secondary objectives were to assess whether EMDR therapy was effective to improve depressive or anxious comorbid symptoms.

**Methods:** We conducted a thorough systematic search of studies published until January 2017, using PubMed, Medline, Scopus, and ScienceDirect as databases. All randomized controlled trials with an EMDR group condition compared to a control group, such as treatment as usual or another psychological treatment, were included. Meta-analysis was conducted with MetaNSUE to avoid biases related to missing information.

**Results:** Eight studies (*n* = 295) met our inclusion criteria. EMDR therapy was superior to waitlist/placebo conditions and showed comparable efficacy to cognitive behavior therapy (CBT) in reducing post-traumatic and anxiety symptoms. A similar but non-statistically significant trend was observed for depressive symptoms. Exploratory subgroup analyses showed that effects might be smaller in studies that included more males and in more recent studies.

**Conclusion:** Despite the small number of publications, the obtained results suggest that EMDR therapy could be a promising psychotherapeutic approach for the treatment of PTSD and comorbid symptoms in young individuals. However, further research with larger samples is needed to confirm these preliminary results as well as to analyze differences in the efficacy of EMDR therapy versus CBT.

## Introduction

According to the fifth edition of the Diagnostic and Statistical Manual of Mental Disorders (DSM-5) (APA, 2013), post-traumatic stress disorder (PTSD) is an anxiety disorder that can appear after an encounter with an unexpected traumatic event and can affect adults, adolescents, and children. The impact of an adverse life event with its negative effects will differ from one population to another depending on a number of factors such as the duration and intensity of the stressor, demographic variables, personality traits, and individual perception (Javidi and Yadollahie, 2012). Furthermore, when focusing on children and adolescents, the level of help and support given by the primary caregivers toward the victims, also plays an essential role in the potential negative consequences of the traumatic event (Javidi and Yadollahie, 2012). The variability of all these factors may be one of the reasons that contribute to the inconclusive evidence in PTSD rates, especially in children (Rodenburg et al., 2009); however, a recent meta-analysis revealed a prevalence around 16% in this population (Morina et al., 2016). Epidemiological studies show that the highest risk period for exposure to many potentially traumatic events is during adolescence, which include interpersonal violence and accidents or injuries among others (McLaughlin et al., 2013). Children, however, can also suffer highly stressful events like domestic violence, physical and/or sexual abuse, neglect, or chronic illnesses which can contribute to the development of a PTSD with or without a comorbid psychiatric disorder (Luthra et al., 2009). In fact, several studies have supported the hypothesis that the exposure to early stressful life events is associated with an increased vulnerability to major psychiatric disorders in adulthood including PTSD, personality disorders, substance use disorders, unipolar depression, bipolar disorder, and schizophrenia (Mclaughlin et al., 2012). Interestingly, there is also an increased risk of somatic illnesses such as obesity, migraines, cardiovascular disease, and diabetes (Javidi and Yadollahie, 2012; Nemeroff, 2016). Furthermore, it has been observed that patients with mood and anxiety disorders with a history of child abuse and neglect, show a worse prognosis of their mental health disorders and have a worse response to pharmacotherapy and/or psychotherapy (Nemeroff, 2016). Therefore, in light of the foregoing, the exposure to early life stressful events can be considered as a major risk factor for mental disorders, hence a rapid trauma orientated intervention is essential to prevent these adverse long term effects. This is especially true in children and adolescents due to their greater vulnerability during brain maturation.

To date, different forms of interventions for childhood PTSD have been used, including pharmacological agents such as tricyclic antidepressants, sertraline, or propranolol. Unfortunately, two systematic reviews concluded that these might be helpful in individual cases but the scientific support for the use of psychopharmacological interventions as a first-line treatment in PTSD in children is currently insufficient (Strawn et al., 2010; Keeshin and Strawn, 2014). Therefore, psychological interventions are the mainstay of treatment in traumatized children and adolescents. International guidelines, supported by several studies, recommend trauma-focused cognitive behavior therapy (TF-CBT) for the treatment of PTSD due to its efficacy to reduce PTSD symptoms and to improve a wide range of other mental health symptoms (Diehle et al., 2015; Morina et al., 2016). However, about a 16–40% of the treated children, continue to fulfill diagnostic criteria for PTSD after treatment (Diehle et al., 2015). Other approaches such as the prolonged exposure for adolescents (PE-A), the narrative exposure therapy (KIDNET), the child–parent psychotherapy (CPP) and the cognitive behavioral interventions for trauma in schools (CBITS), show some evidence of beneficial effects, but conclusions are not concise due an insufficient number of studies (Keeshin and Strawn, 2014; Morina et al., 2016).

A further form of trauma orientated therapy is eye movement desensitization and reprocessing (EMDR) therapy, which has been increasingly used in PTSD and has obtained promising results. This psychotherapeutic approach was developed in the late 80ies by Francine Shapiro. It is an eight-phase treatment approach based on a standardized protocol. Briefly, it consists of history taking, preparation, assessment, desensitization, installation, body scan, closure, and reassessment. This protocol facilitates a comprehensive evaluation of the traumatic memory picture, client preparation, and processing of (a) past traumatic events, (b) current disturbing situations, and (c) future challenges (Shapiro, 2014). One of the components used during the reprocessing phases, and considered as a key element in this therapy, is the bilateral stimulation by saccadic eye movements, tapping, or ear tones. The goal of EMDR therapy is to achieve an adequate processing of the negative experiences and to create new adaptive information. Its effectiveness for the treatment of PTSD in adults has been well-established by several independent meta-analysis (Davidson and Parker, 2001; Seidler and Wagner, 2006; Chen et al., 2014, 2015; Cusack et al., 2016). Numerous organizations, including the American Psychiatric Association, Department of Defense, and World Health Organization, recommend EMDR as an effective treatment for trauma victims (Shapiro, 2014). In the last decade, the number of the studies that have evaluated the efficacy of the EMDR in children or adolescents with PTSD has increased. To date, a meta-analysis carried out by Rodenburg et al. (2009) has analyzed before the efficacy of EMDR in children. This meta-analysis included seven randomized controlled trials with a total sample of 109 children treated with EMDR therapy and 100 children in control conditions. The authors concluded that children receiving EMDR therapy benefited from the intervention and results suggested a small but significant advantage over CBT (Rodenburg et al., 2009). A further meta-analysis compared the evidence of various interventions, including EMDR, focused on man-made and natural disasters and found comparable positive effects of all interventions (Brown et al., 2017).

As new studies have been published, the principal aim of our meta-analysis of RCTs was to update the evidence of the efficacy of EMDR for the treatment of PTSD symptoms in children and adolescents. Secondarily, we also analyzed the effect of EMDR therapy on comorbid depressive and anxious symptoms.

## Methods

The meta-analysis was conducted using the Preferred Reporting Items for Systematic Reviews and Meta-Analyses (PRISMA) checklist and protocol outlined by the PRISMA Group (see Supplementary Table 1) (Moher et al., 2014).

### Protocol and Registration

The protocol of this meta-analysis was registered with the International Prospective Register for Systematic Reviews (PROSPERO) (number CRD42017058769, available at www.crd.york.ac.uk/PROSPERO).

### Eligibility Criteria

Criteria for inclusion for the meta-analysis were as follows: (a) studies that included children or adolescents who had suffered traumatic events and presented symptoms or a PTSD diagnosis; (b) studies that reported results of a RCT evaluating the efficacy of EMDR therapy against a control group, such as treatment as usual, waiting list or another psychological treatment; (c) studies that contained statistics and sufficient data for analyses.

### Information Sources

Using PubMed, Medline, Scopus, and ScienceDirect two of the authors (AMA and ASE) conducted an independent systematic literature search to identify studies published until January 31th 2017 that used EMDR therapy for children or adolescent with trauma caused symptoms or PTSD diagnoses. Furthermore, manual searches of the references list of the previous meta-analysis and the retrieved articles were carried out.

### Search

The search terms were selected from the thesaurus of the National Library of Medicine (Medical Subject Heading Terms, MeSH) and the American Psychological Association (Psychological Index Terms) and included the terms ‘post-traumatic stress disorder,’ ‘PTSD,’ ‘psychological trauma,’ ‘EMDR,’ ‘eye movement desensitization reprocessing therapy,’ ‘children,’ ‘child^∗^,’ and ‘adolescent.’ The final search equation was defined using the Boolean connectors ‘AND’ and ‘OR’ following the formulation ‘post-traumatic stress disorder’ OR ‘PTSD’ OR ‘psychological trauma’) AND (‘EMDR’ OR ‘eye movements reprocessing therapy’) AND (‘children’ OR ‘child^∗^’ OR ‘adolescent’).

### Study Selection

After removing duplicates, AMA and ASE independently screened titles and abstracts and excluded studies that were considered non-pertinent. The final list was accepted by both authors. If inclusion criteria were met, the full text article was retrieved and screened to evaluate the available data for the analysis. Authors of the studies were contacted in case of any doubt (e.g., regarding the randomized process).

### Data Collection Process

Data extraction was independently performed by two authors (DT and ASE). Disagreements were resolved via discussion with a third author (JR) until consensus was reached.

### Data Items

For each article, we recorded the pre-treatment and post-treatment means and standard deviations of the symptoms measures, as well as the effect size of the between-group differences in the pre–post change of these measures. Related statistics (e.g., *t*-values) were also recorded to estimate missing information. PTSD and symptoms related to psychological trauma had been measured with the Peen Inventory for PTSD (Hammaiberg, 1992), the child reaction index (CRI) (Pynoos et al., 1987), the child report of post-traumatic symptoms (CROPS) (Greenwald and Rubin, 1999), the post-traumatic stress symptom scale for children (PTSS-C scale) (Ahmad et al., 2000), the child post-traumatic stress – reaction index (Child PTS-RI) (Frederick et al., 1992) and the Clinician-Administered PTSD Scale for Children and Adolescents (CAPS-CA) (Nader et al., 1996). Depressive and anxiety symptoms had been measured with the Beck depression inventory (BECK) (Beck and Steer, 1993), the children’s depression inventory (CDI) (Kovacs, 1992), the children’s depression scale (CDS) (Lang and Tisher, 1983), the Depression Self Rating Scale (DSRS) (Birlenson, 1981), the Revised Child Anxiety and Depression Scale (RCADS) (Chorpita et al., 2000), the state-trait anxiety inventory (STAI) (Spielberg et al., 1983), the revised children’s manifest anxiety scale (RCMAS) (Reynolds and Richmond, 1985) and the multidimensional anxiety scale for children (MASC) (March et al., 1996).

The following variables were also recorded: year of publication, sample size, participant’s gender distribution, age, comorbid diagnoses (confirmed by clinical interview/clinician assessment), content of the active treatment and control conditions, treatment dose (operationalized as the number of therapy sessions and therapy hours provided), number of patients who dropped out of treatment during the treatment phase, and other clinical and methodological items objectively used to calculate the quality score of each study (see below).

### Risk of Bias in Individual Studies

As recommended by the Cochrane Group (Higgins et al., 2011) we did not search for unpublished data to avoid the inevitable bias caused by dependence on investigators agreeing to provide data from unpublished studies. Included studies were assessed across six domains: adequate sequence generation, allocation concealment, outcome assessment blinding, management of incomplete outcome data, selective reporting and overall risk of bias. Each study was scored using three-item scale: low, high, or unclear risk of bias.

### Quality of Individual Studies

In addition to checking the risk of bias of each study, we assessed their quality using the Jadad scale for randomized controlled trials (Jadad et al., 1996) through three domains: random assignment, double blinding, and the flow of patients. Each study was scored using a range from 0 to 5.

### Summary Measures

Effect size of the difference in severity decrease between groups (Cohen’s delta, i.e., the standardized difference in mean decrease) was directly retrieved from the papers or derived from the reported statistics. Missing data were multiply imputed when possible using the MetaNSUE approach (Radua et al., 2015).

### Synthesis of Results

All effect sizes were corrected for small sample size (Hedges and Olkin, 1985) and separately meta-analyzed for each set of the multiple imputations using random-effects models, which take both intra-study and between-study variability into account. The latter, also called “heterogeneity,” was estimated with the optimal restricted maximum likelihood (REML) technique (Viechtbauer, 2005).

Consistency of these differences was assessed by: (a) estimating the percentage of variability due to between-study heterogeneity (*I*^2^) and the probability that this is statistically significantly different from 0% (so-called “Q test,” but using an F statistic due to the multiple imputations); and (b) conducting leave-one-out jack-knife analyses (i.e., iteratively repeating the meta-analysis with all studies but one).

The multiple results originated from the different imputation sets were pooled taking imputation variability into account (Radua et al., 2015).

Separate meta-analyses were also conducted for post-traumatic, anxiety, and depression symptoms.

### Drop-out Analysis

Possible differences in the number of patients who dropped out prematurely from treatment were investigated via a meta-analysis of the (logarithm-transformed) relative risk that a patient dropped out from the CBT group (as compared to the control group).

### Risk of Bias across Studies

Potential bias was assessed by meta-regressing the effect sizes by their standard errors in order to detect whether studies with larger standard errors (due to e.g., small sample sizes) report larger effect sizes.

### Analysis of Subgroups

For exploratory purposes, separate analysis were conducted for studies with <50% vs. >50% females, for studies comparing EMDR to CBT vs. other control groups, for studies applying <5 sessions vs. >5 sessions, for studies using <4 h per session vs. ≥4 h per session, for studies evaluating the patients before 3 months vs. at least 3 months after, for studies using an intention-to-treat analysis vs. studies using a per protocol analysis, and for studies published before 2008 vs. from 2008 onward. Subgroup analyses were only conducted when the two complementary subgroups included at least two studies each. No formal comparisons were conducted between each pair of subgroups due to the small numbers of studies.

### Role of the Funding Source

The funder had no role in study design, data collection, data analysis, data interpretation, or writing of the paper.

## Results

### Study Selection and Study Characteristics

**FIGURE 1:**
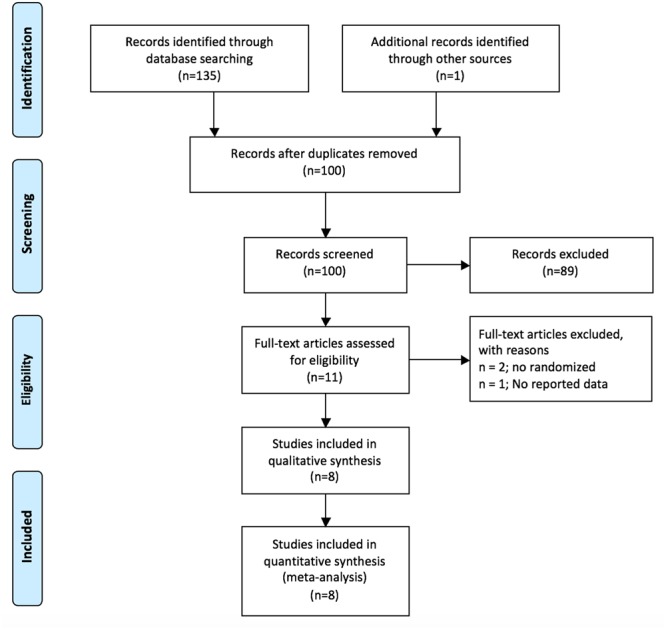
Flow diagram of excluded and included studies according to the PRISMA guide.

The PRISMA flowchart is shown in **Figure 1**. Eleven studies out of a total 136 were initially screened and analyzed for eligibility, leading to a total of eight final studies included in the review, comprising 295 participants with PTSD or trauma caused symptoms. All studies but one (Scheck et al., 1998) included exclusively children and adolescents with PTSD or trauma caused symptoms and involved individually delivered face-to-face EMDR sessions compared to no treatment (Soberman et al., 2002), pure waiting list (Chemtob et al., 2002; Ahmad et al., 2007; Kemp et al., 2010) active listening control (Scheck et al., 1998) or CBT (Jaberghaderi et al., 2004; de Roos et al., 2011; Diehle et al., 2015) (see **Table 1**).

**Table 1:** Studies included in the meta-analysis.

Study	N		Age (years)	Females	Control group	Randomized	Blinded	Sessions		Months post	ITT analysis
	EMDR	Control						N	Hours		
Scheck et al., 1998	34	33	20.9	100%	ALC	Yes	Yes	2	3	3	No
Chemtob et al., 2002	19	15	8.4	NA	WL	Yes	Yes	3	NA	6	No
Jaberghaderi et al., 2004	10	9	12.5 ^(a)^	100%	CBT	Yes	Yes	6.1	3	0.5	No
Ahmad et al., 2007	17	16	9.9	60.6%	WL	Yes	Yes	5.9	4.5	2	Yes
Soberman et al., 2002	14	15	13.0 ^(a)^	0%	TAU	Yes	Yes	3	3	0.5	No
Kemp et al., 2010	13	14	8.9	44.4%	WL	Yes	No	4	4	12	No
de Roos et al., 2011 ^(b)^	26	26	11.8	44.2%	CBT	Yes	Yes	3.2	3	3	Yes
Diehle et al., 2015	25	23	12.9	62.5%	CBT	Yes	Yes	8	8	NA	Yes

*^(a)^Average of age range; ^(b)^only the ≥7-year old subsample was analyzed, values here are for the whole sample with the exception of the mean age which has been scaled to the ≥7-year old subsample. ALC, active listening control; CBT, cognitive behavioral therapy; EMDR, eye movement desensitization and reprocessing; ITT, intention to treat; NA, not available; TAU, treatment as usual; WL, waiting list.*

### Risk of Bias within Studies

**Table 2** provides data on the risk of bias measured using the Cochrane Collaboration’s Tool for Assessing Risk of Bias. Of the analyzed studies, five had unclear risk (Scheck et al., 1998; Chemtob et al., 2002; Soberman et al., 2002; Ahmad et al., 2007; Kemp et al., 2010) and three were considered to have low risk of bias (Jaberghaderi et al., 2004; de Roos et al., 2011; Diehle et al., 2015).

**Table 2:** Indicators of study quality based on the Cochrane collaboration’s tool for assessing risk bias (Higgins et al., 2011).

Study (chronological order)	Adequate sequence generation	Allocation concealment	Blinding (outcome assessment)	Incomplete outcome data addressed	Free of selective reporting	Overall risk of bias
Scheck et al., 1998	Low	Low	Low	Low	Unclear	Unclear
Chemtob et al., 2002	Unclear	Unclear	Low	Low	Low	Unclear
Soberman et al., 2002	Unclear	Unclear	Low	Low	Low	Unclear
Jaberghaderi et al., 2004	Low	Low	Low	Low	Low	Low
Ahmad et al., 2007	Unclear	Unclear	Low	Low	Low	Unclear
Kemp et al., 2010	Unclear	Unclear	Unclear	Low	Low	Unclear
de Roos et al., 2011	Low	Low	Low	Low	Low	Low
Diehle et al., 2015	Low	Low	Low	Low	Low	Low

### Quality of Individual Studies

**Table 3** provides data on the quality of the studies using the Jadad Scale (0–5 points). Of the analyzed studies, one scored 2 points (Kemp et al., 2010), another 3 points (Soberman et al., 2002), two scored 4 points (Chemtob et al., 2002; Ahmad et al., 2007) and the rest of the studies scored 5 points (Scheck et al., 1998; Jaberghaderi et al., 2004; de Roos et al., 2011; Diehle et al., 2015).

**Table 3:** Jadad scale for randomized controlled trials (Jadad et al., 1996).

Study (chronological order)	Randomization	Blinding	An account of all patients	Total score (maximum points = 5)
Scheck et al., 1998	2	2	1	5
Chemtob et al., 2002	1	2	1	4
Soberman et al., 2002	1	1	1	3
Jaberghaderi et al., 2004	2	2	1	5
Ahmad et al., 2007	1	2	1	4
Kemp et al., 2010	1	0	1	2
de Roos et al., 2011	2	2	1	5
Diehle et al., 2015	2	2	1	5

### Post-traumatic Symptoms

The meta-analysis of post-traumatic symptoms included all studies, six of them with known effects and two with unknown non-statistically-significant effects. EMDR therapy decreased trauma-associated symptoms in a significant way (*d* = -0.49, *z* = -2.5, *p* = 0.013, 95% CI = -0.87 to -0.10). This analysis showed moderate but non-statistically-significant heterogeneity (I2 = 52%, *p* = 0.072), without potential publication bias (*p* = 0.860). The Jackknife analysis suggested that the meta-analysis was not statistically significant after exclusion of either the study by Scheck et al. (1998) or the study by Ahmad et al. (2007) though effect sizes were still similar (from -0.58 to -0.36) (see **Table 4**).

**Table 4:** Meta-analysis of post-traumatic, anxiety, and depression symptoms.

	Post-traumatic		Anxiety		Depression	
	Effect size	*p*-value	Effect size	*p*-value	Effect size	*p*-value
All studies	–0.49	0.013	–0.44	0.006	–0.27	0.118
Jackknife, study discarded:						
Scheck et al., 1998^∗^	–0.46	0.057	–0.39	0.057	–0.11	0.593
Chemtob et al., 2002	–0.36	0.022	–0.37	0.043	–0.19	0.381
Jaberghaderi et al., 2004	–0.53	0.014				
Ahmad et al., 2007	–0.40	0.052				
Soberman et al., 2002	–0.51	0.014				
Kemp et al., 2010^∗∗^	–0.51	0.016	–0.46	0.005	–0.28	0.134
de Roos et al., 2011	–0.58	0.005	–0.55	0.004	–0.40	0.037
Diehle et al., 2015	–0.57	0.010	–0.43	0.015	–0.33	0.078
Subgroup analyses:						
<50% females	–0.03	0.908	–0.12	0.694	0.04	0.883
>50% females	–0.48	0.016	–0.52	0.023	–0.31	0.338
Compared to CBT	–0.09	0.636	–0.25	0.336	0.08	0.747
Compared to other	–0.79	<0.001	–0.56	0.009	–0.51	0.014
<5 sessions	–0.52	0.068				
>5 sessions	–0.43	0.160				
<4 h per session	–0.30	0.135	–0.37	0.068	–0.27	0.379
≥4 h per session	–0.46	0.138	–0.36	0.332	0.11	0.765
Post < 3 months	–0.60	0.060				
Post ≥ 3 months	–0.58	0.080				
ITT analysis	–0.36	0.214	–0.25	0.336	0.08	0.747
Per protocol analysis	–0.60	0.025	–0.56	0.009	–0.51	0.016
Published before 2008	–0.84	0.001	–0.61	0.005	–0.55	0.010
Published from 2008	–0.09	0.662	–0.23	0.355	0.08	0.729

*Subgroup analyses only conducted when the two complementary subgroups included at least two studies each. ^∗^ The only study included adults in the sample; ^∗∗^ the only non-blinded study. CBT, cognitive behavioral therapy; ITT, intention to treat.*

### Anxiety Symptoms

The meta-analysis of anxiety symptoms included five studies (Scheck et al., 1998; Chemtob et al., 2002; Kemp et al., 2010; de Roos et al., 2011; Diehle et al., 2015). Four of them had known effects and one had unknown non-statistically-significant effects. EMDR therapy proved to decrease significantly anxiety symptoms (*d* = -0.44, *z* = -2.7, *p* = 0.006, 95% CI = -0.76 to -0.13). Again, this analysis showed no heterogeneity (I2 = 1%, *p* = 0.747) and no potential publication bias (*p* = 0.977). Jackknife analysis showed that the meta-analysis was not statistically significant after exclusion of the study by Scheck et al. (1998), though effect sizes were still similar (from -0.55 to -0.37) (see **Table 4**).

### Depression Symptoms

The meta-analysis of depressive symptoms included five studies (Scheck et al., 1998; Chemtob et al., 2002; Kemp et al., 2010; de Roos et al., 2011; Diehle et al., 2015). Four of the studies had known effects and one had unknown non-statistically-significant effects. EMDR therapy did not show a statistically significant decrease of depressive symptoms (*d* = -0.27, *z* = -1.6, *p* = 0.118, 95% CI = -0.61 to 0.07). This analysis showed no heterogeneity (I2 = 11%, *p* = 0.416), and no potential publication bias (*p* = 0.366). Jackknife analysis showed effect sizes in the range (-0.40, -0.11) (see **Table 4**).

### Drop-out Analysis

No differences in the number of drop-out patients were detected between the EMDR and control groups (relative risk = 1.04, 95% CI = 0.97 to 1.12; *p* = 0.287).

### Analysis of Subgroups

For post-traumatic symptoms, subgroup analyses showed that the effect size was nearly null (a) in studies that included mostly male patients (*d* = -0.03), (b) in studies that compared EMDR to CBT (*d* = -0.09) and (c) in studies published from 2008 onward (*d* = -0.09) (see **Table 4**).

For anxiety symptoms, subgroup analyses suggested that the effect size was small (a) in studies that included mostly male patients (*d* = -0.12), (b) in studies published from 2008 onward (*d* = -0.23), (c) in studies that compared EMDR therapy to CBT (*d* = -0.25) and (d) in studies that had applied an intention to treat analysis (*d* = -0.25) (see **Table 4**).

Finally, for depressive symptoms, subgroup analyses showed that the effect size was nearly null (a) in studies that included mostly male patients (*d* = 0.04), (b) in studies that compared EMDR therapy to CBT (*d* = 0.08), (c) in studies that had applied an intention to treat analysis (*d* = 0.08), (d) in studies published from 2008 onward (*d* = 0.08) and (e) in studies that had applied four or more sessions (*d* = 0.11) (see **Table 4**).

## Discussion

This is the third meta-analysis that explores the evidence of the efficacy of EMDR to treat trauma-associated symptoms in children and adolescents and the first one to assess its efficacy in depressive and anxiety symptoms associated with traumatic events. The main result of this meta-analysis is that patients treated with EMDR therapy present a reduction of their trauma-associated symptoms as compared to patients in the respective control conditions, this effect was also observed for comorbid anxiety symptoms (*d* = -0.49 and -0.44, *p* < 0.013). A similar but non-statistically-significant trend was observed for trauma-associated depressive symptoms (*d* = -0.27, *p* = 0.118).

Our results are similar to the previous meta-analysis carried out by Rodenburg et al. (2009), who also found that children treated with EMDR benefited from the treatment. That meta-analysis also found that EMDR was more effective than CBT (*d* = 0.56, *p* < 0.001) (Rodenburg et al., 2009), a finding that has not been detected in our updated meta-analysis. However, both meta-analyses are in line with recent meta-analytic studies analyzing EMDR therapy in adult samples, which showed that this psychotherapeutic approach reduces the symptoms of PTSD (Chen et al., 2014; Cusack et al., 2016) and is at least as effective as other techniques such as CBT (Davidson and Parker, 2001; Seidler and Wagner, 2006; Cusack et al., 2016).

Regarding comorbid depressive and anxiety symptoms, our meta-analysis is also in line with a meta-analysis carried out by Chen et al. (2014), which showed that EMDR therapy reduced depression and anxiety symptoms in adults with PTSD (Chen et al., 2014). The results of our meta-analysis reached statistical significance for the reduction of anxious symptoms but not for the reduction of depressive symptoms. However, the lack of statistical significance could be due to the small number of studies (*n* = 5) included in this analysis. More studies are needed to confirm these preliminary results.

Complementary analyses did not detect potential reporting bias, and the effect size was relatively similar throughout the jackknife iterations. In addition, no differences in the number of drop-out patients were detected between the EMDR and control groups. Conversely, exploratory subgroup analyses showed that the effect size was small or nearly null when studies with mostly male patients, comparative studies of EMDR to CBT, or studies published from 2008 onward were included only. The lack of effect of EMDR therapy in male patients is interesting, as current evidence suggests that girls are more likely to develop PTSD than boys (Alisic et al., 2014), especially when they have suffered interpersonal trauma. Regarding the lack of differences in the efficacy of EMDR therapy compared to CBT, evidence in adults suggests -as stated before- that both approaches to treat PTSD are comparable (Davidson and Parker, 2001; Seidler and Wagner, 2006; Chen et al., 2015). However, subgroup analyses must be understood as exploratory given the small number of studies included in each subgroup. Our data are also in line with the second meta-analysis which included 34 studies and examined the effectiveness of EMDR, CBT, KIDNET and classroom-based interventions in children and adolescents after man-made and natural disasters (Brown et al., 2017). The authors did not reveal significant differences in pre–post scores within interventions. Importantly, six of the studies included in the meta-analysis applied group EMDR instead of individual sessions, a factor that might have reduced the efficacy of EMDR, as EMDR therapy was originally developed as an individual psychotherapy.

Eye movement desensitization and reprocessing is a complex psychotherapeutic approach that involves behavioral, cognitive, emotional, and psychical components in which each one plays an important role. Saccadic eye movements are elicited mainly to alleviate negative cognition, negative emotion, and unpleasant physical sensations associated with a traumatic memory and to reinforce positive cognition (Coubard, 2016). Despite EMDR has been validated as an effective treatment for PTSD based on controlled clinical research, the scientific community is divided about this intervention because its underlying neural mechanism is unknown (Coubard, 2016). Currently, several hypotheses have been proposed to explain the effectiveness of EMDR, related to orienting response, interhemispheric connection, visuospatial sketchpad and rapid eye movement (REM)-like movement (Novo et al., 2016), but none of them is sufficient to explain the effectiveness of EMDR.

The research about EMDR is still in its infancy, and more research is needed to understand better its mechanism of action and the underlying neural mechanism. More studies are also needed to confirm the preliminary results about the effectiveness of this psychotherapeutic approach in children and adolescents suffering from PTSD.

## Limitations

Several limitations have to be taken into account before translating these results into clinical settings. First, the small number of studies included in this meta-analysis might have prevented the detection of some effects, such as the reduction of depressive symptoms. We included RCT only and discarded other types such as non-randomized, observational or case studies, which decreased statistical power but avoided possible biases. Secondly, the studies included in the meta-analysis used different control conditions, which reflects the heterogeneity of this field. Three studies used pure waiting list, three used CBT, one active listening and another one did not use any active control condition. Also, the number of EMDR sessions that participants received in some studies was relatively low, for instance patients only received two sessions in the study performed by Scheck et al. (1998). This could be insufficient bearing in mind the eight phases of the standard protocol and the complexity of trauma-associated and comorbid symptoms. Finally, the small number of studies prevented a multivariate analysis to discard whether the factors analyzed in the subgroup analyses may be confounding each other. Therefore, no strong conclusions should be taken regarding the effects of gender, the comparison with CBT or the publication year.

## Conclusion

Despite the small number of publications, the results of this meta-analysis suggests that EMDR could be a promising psychotherapeutic approach for the treatment of PTSD and anxiety symptoms in children and adolescents. However, further research with larger samples is needed to confirm these preliminary results.

## Author Contributions

AM-A and BA had the idea of the project. AM-A and AS-E conducted the systematic literature search to identify studies published and the screening of the manuscripts. DT and AS-E performed data extraction. JR conducted the statistical analysis. AM-A, AV-G, and JR wrote the first draft of the manuscript. AM-A, DT, AV-G, AS-E, VP, BA, and JR contributed to the revisions and modifications of the manuscript and all have approved the final version.

## Conflict of Interest Statement

BA has been invited as speaker to various national and international congresses of EMDR. The other authors declare that the research was conducted in the absence of any commercial or financial relationships that could be construed as a potential conflict of interest.
